# Dual-Model GWAS Analysis and Genomic Selection of Maize Flowering Time-Related Traits

**DOI:** 10.3390/genes15060740

**Published:** 2024-06-04

**Authors:** Zehui Fan, Shaohang Lin, Jiale Jiang, Yukang Zeng, Yao Meng, Jiaojiao Ren, Penghao Wu

**Affiliations:** College of Agronomy, Xinjiang Agricultural University, Urumqi 830052, China; 17860727550@163.com (Z.F.); 15687041941@163.com (S.L.); 17591666601@163.com (J.J.); 15727549889@163.com (Y.Z.); qnbz2250@163.com (Y.M.); renjiaojiao789@sina.com (J.R.)

**Keywords:** maize, flowering time, genome-wide association analysis, candidate genes, genomic selection, prediction accuracy

## Abstract

An appropriate flowering period is an important selection criterion in maize breeding. It plays a crucial role in the ecological adaptability of maize varieties. To explore the genetic basis of flowering time, GWAS and GS analyses were conducted using an associating panel consisting of 379 multi-parent DH lines. The DH population was phenotyped for days to tasseling (DTT), days to pollen-shedding (DTP), and days to silking (DTS) in different environments. The heritability was 82.75%, 86.09%, and 85.26% for DTT, DTP, and DTS, respectively. The GWAS analysis with the FarmCPU model identified 10 single-nucleotide polymorphisms (SNPs) distributed on chromosomes 3, 8, 9, and 10 that were significantly associated with flowering time-related traits. The GWAS analysis with the BLINK model identified seven SNPs distributed on chromosomes 1, 3, 8, 9, and 10 that were significantly associated with flowering time-related traits. Three SNPs 3_198946071, 9_146646966, and 9_152140631 showed a pleiotropic effect, indicating a significant genetic correlation between DTT, DTP, and DTS. A total of 24 candidate genes were detected. A relatively high prediction accuracy was achieved with 100 significantly associated SNPs detected from GWAS, and the optimal training population size was 70%. This study provides a better understanding of the genetic architecture of flowering time-related traits and provides an optimal strategy for GS.

## 1. Introduction

Maize (*Zea mays* L.) is the highest-yielding cereal crop in the world, playing a crucial role in food security [1]. The flowering time of maize is a crucial factor influencing the adaptability of maize varieties to regional environments and has been considered a critical selection criterion in crop breeding [2]. Days to tasseling (DTT), days to pollen-shedding (DTP), and days to silking (DTS) are important flowering time-related traits in maize. Flowering time-related traits in maize were typically quantitative traits regulated by many genes. Shi et al. reported that the broad-sense heritability across two environments of DTT, DTP, and DTS were 76.20%, 67.78%, and 68.03%, respectively [3]. The phenotypic variations in flowering time-related traits are mainly controlled by genetic factors [4].

A large number of QTLs have been identified for maize flowering time-related traits [5,6,7]. In the study of Liu et al. (2016), six QTLs on chromosomes 1, 5, 9, and 10 were detected for DTT, explaining 3.42–11.79% of the phenotypic variation; twenty-one QTLs on chromosomes 1, 5, 6, 7, 8, 9, and 10 were detected for DTP, explaining 0.8–12.95% of the phenotypic variation; twenty-two QTLs on chromosomes 1, 3, 4, 5, 6,7, 9, and 10 were detected for DTS, explaining 1.77–13.47% of the phenotypic variation; and seventeen QTLs on chromosomes 1, 3, 4, 5, 6, 7, 8, and 10 were detected for anthesis-silking interval (ASI), explaining 0.38–13% of the phenotypic variation [5]. In the RIL population of B73 × Abe2, eight QTLs with the phenotypic variation explained (PVE) ranging from 1.92 to 17.28%, thirteen QTLs with the PVE ranging from 2.09 to 13.08%, and fifteen QTLs with the PVE ranging from 2.28 to 14.87% were identified for days to heading (DTH), DTS, and days to anthesis (DTA), respectively (Shi et al., 2022) [3]. Wu et al. identified eleven QTLs associated with DTT explaining 11.4–32.5% of the phenotypic variation, four QTLs associated with DTP explaining 2.4–35.4% of the phenotypic variation, and six QTLs associated with DTT explaining 7.8–32.6% of the phenotypic variation [6]. Li et al. identified four and three QTLs associated with DTA and DTS explaining 35.37% and 34.22% of the total phenotypic variation, respectively [7].

GWAS is a powerful tool for exploring the genetic architecture of complex traits and candidate genes. In the GWAS panel consisting of 226 maize inbred lines, 82 SNPs and 117 candidate genes were identified for DTT, DTP, and DTS (Wu et al., 2023) [6]. Some SNPs were significantly associated with two or three-flowering time-related traits, indicating a significant genetic correlation between flowering time-related traits. Li et al. (2016) identified nearly 1000 flowering time-associated SNPs and 220 candidate genes in an extremely large multi-genetic background population [8]. All the studies are of great significance for understanding the genetic basis of maize flowering time, which is still unclear. Exploring new genetic loci and candidate genes is necessary for revealing the genetic regulation of maize flowering time.

To improve the breeding efficiency and cost-effectiveness, Meuwissen et al. introduced the concept of genomic selection (GS), where genome-wide markers were used for selection [9]. A training population with phenotype and genotype data was used to estimate the genomic estimated breeding values (GEBVs) of individuals in the prediction population with genotype data. The accuracy of the prediction is measured by the correlation between GEBVs and true breeding values. Yuan et al. (2019) conducted GS of anthesis date and ASI under well-watered (WW), drought stress (DS), heat stress (HS), and combined drought and heat stress (DTS) management conditions. The prediction accuracies were 0.64, 0.62, 0.45, and 0.51 for anthesis date, and 0.40, 0.55, 0.13, and 0.29 for ASI under WW, DS, HS, and DHS condition, respectively [10].

Previous studies have indicated that the prediction ability of GS depends on the prediction accuracy, which is influenced by various factors, including the trait heritability, prediction models, environmental and seasonal factors, training population size, the genetic relationship between training and prediction sets, marker density, and marker quality [11,12,13,14,15]. For traits affected by multiple small-effect QTL, GBLUP or RR-BLUP may achieve better prediction accuracy compared to the Bayesian models [15]. Genomic selection for flowering time-related traits using deep learning models and Bayesian models has been proposed by Mora-Poblete (2023), and deep learning models showed higher prediction consistently [16]. In the study of Beyene et al., the prediction accuracy of anthesis date (AD) was 0.37 and 0.40 under well-watered conditions and water-stressed conditions, respectively [17]. The prediction accuracy of flowering time-related traits reported previously was moderate to low. Efforts should be made to improve the prediction accuracy of flowering time-related traits to improve the breeding efficiency.

In the present study, 379 multi-parent doubled haploid (DH) lines were phenotyped for DTT, DTP, and DTS in four different environments and genotyped by the 48 K liquid-phase hybridization probe capture technique for GWAS and GS. This study aims to (1) identify SNPs and putative candidate genes conferring flowering time-related traits by different GWAS models; (2) explore the potential of GS for flowering time-related traits; and (3) estimate the effect of training population size, marker density, and significantly associated SNPs on prediction accuracy.

## 2. Materials and Methods

### 2.1. Plant Materials and Field Trials

In this study, a multi-parent DH population consisting of 379 DH lines was utilized as the association analysis population. The DH lines were developed from 21 maize hybrids (Supplementary Table S1). The experiment was conducted in four different environments, Sangong Town experimental station, Changji City, Xinjiang, China (SG, 87°12′57″ E, 43°56′54″ N) and Dafeng Town experimental station, Hutubi County, Xinjiang, China (DF, 86°34′49″ E, 44°10′47″ N) during the summer of 2022; Ledong experimental station, Hainan Province, China (LD, 108°57′14″ E, 18°27′14″ N) during the winter of 2022; and Qitai County experimental station, Xinjiang, China (QT, 89°44′19″ E, 44°5′77″ N) during the summer of 2023. The experiment was performed using a completely randomized block design with single-row plots. Each row had a length of 2.5 m, row spacing of 0.6 m, and plant spacing of 0.25 m. All field management, including fertilization, irrigation, pest and disease control, and weed management, followed the local standardized field management.

### 2.2. Evaluation and Data Analysis of Flowering Time-Related Traits

Days to tasseling (DTT), days to pollen-shedding (DTP), and days to silking (DTS) were evaluated as the number of dates from planting until half of the individuals in each row reached tasseling, pollen-shedding, and silking. R version 4.3.1 software [18] was employed for descriptive statistical analysis, variance analysis, correlation analysis, and visualization of the phenotypic data related to flowering time-related traits. The “lmer” function in the lme4 package of R was used to calculate variance components, best linear unbiased prediction (BLUP) values, and broad-sense heritability. BLUP values across all four environments were deployed for GWAS and GS. The broad-sense heritability was calculated on an entry-mean basis according to the method described by Hallauer et al. [19].

### 2.3. Genotyping

The DNA extraction and genotyping of the DH population were conducted by China Golden Marker Biotech Co., (Beijing, China). The 48 K liquid-phase hybridization probe capture technique was used. This process involved DNA concentration and quality evaluation, library construction, and sequencing. Clean reads were received after quality control using Trimmomatic-0.36 [20]. BWA 0.7.17 software [21] was used for SNP calling based on the Maize B73_RefGen_v4 reference genome. A total of 1,583,425 SNPs were received and filtered by vcftools (Danecek et al., 2011 [22]). Finally, 134,785 high-quality SNPs with the missing rate (MR) < 20%, and the minor allele frequency (MAF) > 0.05 were retained.

### 2.4. Genome-Wide Association Analysis

Two multi-locus models, fixed and random model circulating probability unification (FarmCPU) and bayesian information and linkage-disequilibrium iteratively nested keyway (BLINK), from the GAPIT version 3.0, were employed for GWAS of flowering time-related traits [23]. The population structure was controlled by the first three PCs. The FarmCPU model effectively controls false positives and reduces false negatives, allowing rapid analysis of large populations with numerous markers [24]. The BLINK model enhances statistical power by eliminating the assumption that causal genes are uniformly distributed in the genome [23]. A Bonferroni-corrected *p*-value of 3.71 × 10^−7^ (0.05/total number of markers) was used as the significance threshold, with a −log10 (*p*-value) of 6.4.

### 2.5. Candidate Gene Discovery

Genes located within 10 kb upstream and downstream of a significant SNP were retrieved as candidate genes based on the B73 RefGen_v4 reference genome on the maizeGDB website (https://www.maizegdb.org/ accessed on 1 September 2023), Candidate genes were annotated with databases of NCBI (https://www.ncbi.nlm.nih.gov/ accessed on 1 September 2023), GO (http://www.geneontology.org/ accessed on 1 September 2023), and the National Center for Bioinformation (https://www.cncb.ac.cn/ accessed on 1 September 2023).

### 2.6. Genomic Selection

Ridge regression best linear unbiased prediction (RR-BLUP) was one of the most commonly used models for GS. The rrBLUP package version 4.6.3 [25] in the R software was used for GS of flowering time-related traits. A k-fold cross-validation with k = 5 was performed 100 times, randomly selecting 20% of the population as the prediction population and the remaining 80% as the training population. The RR-BLUP model was fitted using the training data, and the response variable in the testing set was predicted. The average of prediction accuracy was calculated.

With all 134,785 SNPs, the training population size was set at 10%, 20%, 30%, 40%, 50%, 60%, 70%, 80%, and 90% of the total population, and the remaining population was used as the prediction population for GS. The accuracy of GS for different population sizes was measured as the average of 100 replications.

The 5-fold cross-validation with 100 replications was employed to study the effect of marker density and significant SNPs on GS. The marker density was set to 10, 30, 50, 100, 300, 500, 1000, 3000, and 5000, which were selected randomly. The significant SNP markers identified by two GWAS models were used for GS. The SNPs were ordered according to *p*-value, and 1, 3, 5, 10, 30, 50, 100, 300, and 500 SNPs with the lowest *p*-value were selected for GS. The remaining SNPs were not used for GS. The one-way ANOVA with an LSD multiple comparison test was used to compare the differences between the prediction accuracy, estimated by all the markers and significant SNPs detected by two GWAS models, different training population sizes, and different marker densities.

## 3. Results and Analysis

### 3.1. Descriptive Statistical Analysis of Flowering Time-Related Traits

Descriptive statistical analysis was performed on the phenotypes of single and combined environments (Table 1). ALL three-flowering time-related traits varied among the environments and sufficient variation was observed in each environment. The average DTT was 59.94, 54.63, 56.41, 71.06, and 60.55 days in SG, DF, LD, QT, and combined environments, respectively. The greatest differentiation of DTT was observed in SG, where DTT ranged from 50.00 to 72.00. The smallest differentiation of DTT was observed in DF, where DTT ranged from 50.00 to 62.00.

The average DTP was 64.30, 62.80, 59.80, 72.90, and 65.00 in SG, DF, LD, QT, and combined environments, respectively. The greatest differentiation of DTP was observed in SG and QT, where DTP ranged from 54.0 to 77.0. The smallest differentiation of DTP was observed in combined environments, where DTP ranged from 58.2 to 73.0.

The average DTS was 65.87, 63.73, 61.29, 73.78, and 66.30 in SG, DF, LD, QT, and combined environments, respectively. The greatest differentiation was detected in SG, DF, and QT. The smallest differentiation was observed in combined environments, where DTS ranged from 58.8 to 75.0. The highest average values of DTT, DTP, and DTS were observed in QT. The values of skewness and kurtosis were within the range of −1 to 1, indicating that the three-flowering time-related traits followed a normal distribution.

### 3.2. Analysis of Variance for Flowering Time-Related Traits

A variance analysis was conducted for flowering time-related traits across multiple environments (Table 2). The results indicated that the differences in flowering time-related traits reached extremely significant levels in terms of genotype, environment, and genotype-environment interaction (*p* < 0.001). This suggested that, in addition to being controlled by genotype, the three-flowering time-related traits were also influenced by environmental factors and the interaction between genotype and environment. The heritability of DTT, DTP, and DTS was similar, with values of 82.75%, 86.09%, and 85.26%, respectively. This indicated that the three-flowering time-related traits were primarily under genetic control.

### 3.3. Correlation Analysis of Flowering Time-Related Traits

**Figure 1:**
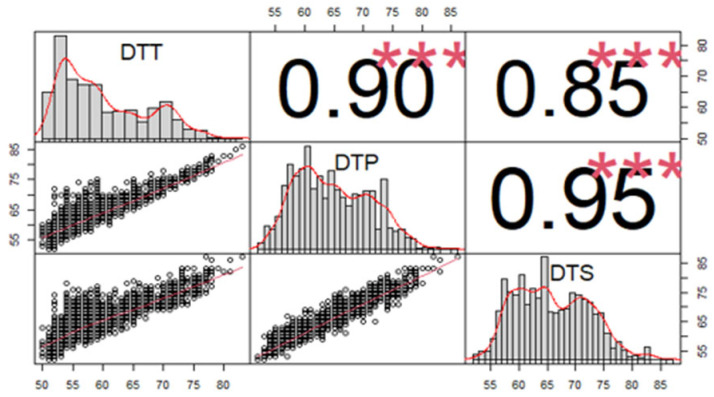
Correlation analysis of flowering time-related traits. DTT: days to tasseling; DTP: days to pollen-shedding; DTS: days to silking. ***: significant at the 0.001 level. The red lines are regression lines.

Correlation analysis was conducted based on the BLUP values of flowering time-related traits (Figure 1). The results showed that there was a highly significant positive correlation between each of the two traits. The correlation coefficient between DTP and DTS was the highest, with an r-value of 0.95, while the correlation coefficient between DTT and DTS was the lowest, with an r-value of 0.88.

### 3.4. Genome-Wide Association Analysis of Flowering Time-Related Traits

**Figure 2:**
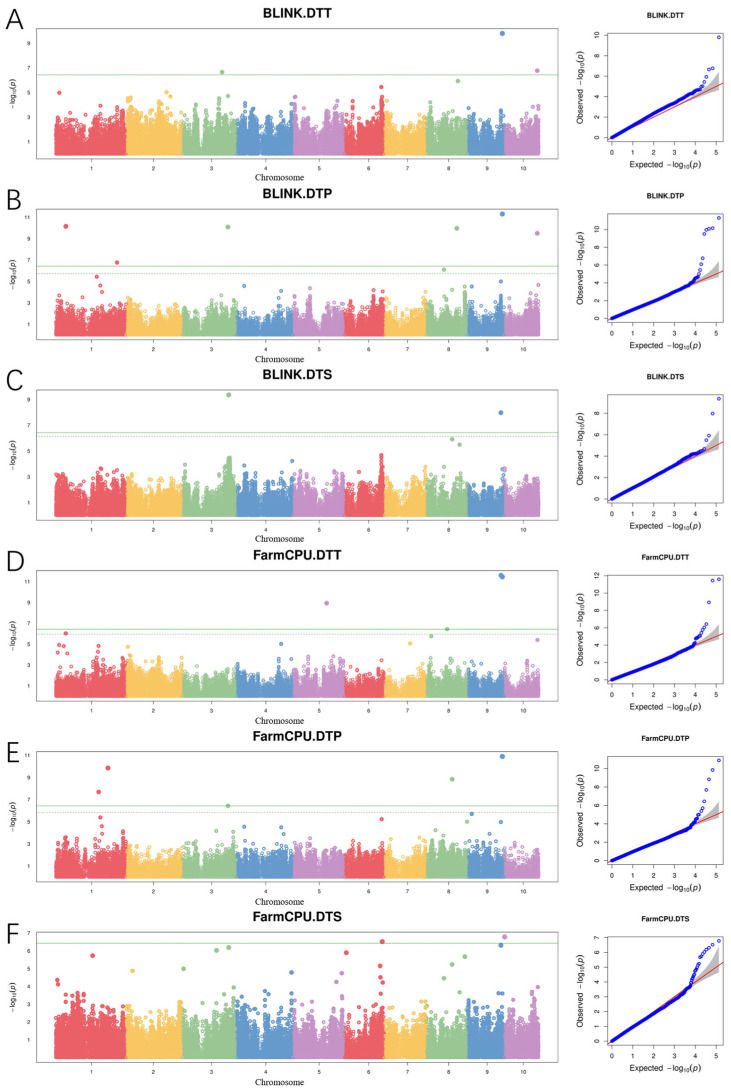
Manhattan and QQ plots of flowering time-related traits by dual-model. (**A**): days to tasseling of Blink model; (**B**): days to pollen-shedding of Blink model; (**C**): days to silking of Blink model; (**D**): days to tasseling of FarmCPU model; (**E**): days to pollen-shedding of FarmCPU model; (**F**): days to silking of FarmCPU model. In the manhattan plots, different colors represent different chromosomes. In the QQ plots, red lines represent that the expected −log_10_(*P*) is equal to the observed −log_10_(*P*).

To accurately identify the significant loci associated with maize flowering time, BLINK, and FarmCPU, two multi-locus models were employed for the GWAS of flowering time-related traits. With the threshold at −log10p > 6.4, the BLINK model detected nine SNPs, including three for DTT, four for DTP, and two for DTS. The FarmCPU model detected thirteen SNPs, including three for DTT, six for DTP, and four for DTS (Table 3, Figure 2).

For DTT, the BLINK model identified three significantly associated SNPs on chromosomes 1, 9, and 10, accounting for 20.3% of phenotypic variation in total. The SNP 1_190275500 located on chromosome 1 had the lowest *p*-value of 5.52 × 10^−11^. The SNP 9_152140631 located on chromosome 9 was a major loci accounting for 16.92% of phenotypic variation. The FarmCPU model detected three SNPs associated with DTT on chromosomes 3, 9, and 10, accounting for 23.42 of phenotypic variation in total. The SNP 9_152140631 with the lowest *p*-value of 7.35 × 10^−15^ had a PVE of 15.53%. It was detected by both models.

For DTP, four significant SNPs located on chromosomes 3, 8, and 9 were identified by the BLINK model. The PVE of each SNP ranged from 2.64 to 12.68%, explaining 23.27% of the phenotypic variance in total. The most significant SNP 9_152140631 explained 12.68% of the phenotypic variance. Six SNP on chromosomes 3, 8, 9, and 10 were detected for DTP by the FarmCPU model. Each SNP explained 0.50 to 5.82% of the phenotypic variance, with a total PVE of 15.99%. SNP 9_152140631 was also identified in the FarmCPU model.

For DTS, two significant SNPs distributed across chromosomes 8 and 9 were identified by the BLINK model, explaining 3.39% and 24.17% of the phenotypic variation. Four SNPs located on chromosomes 3, 8, and 9 were detected by the FarmCPU model. The PVE of each SNP ranged from 2.30 to 8.57%, with a total PVE of 21.29%. SNPs 8_78666793 and 9_15214063 were co-located by both models.

**Figure 3:**
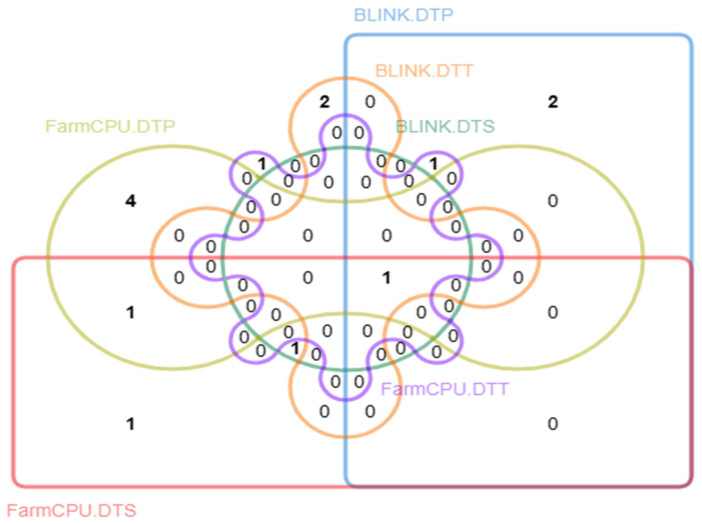
Venn plot and the number of significant SNPs detected by each model.

Four SNPs, 3_198946071, 8_78666793, 9_146646966, and 9_152140631, were repeatedly identified by different models or traits (Figure 3). SNP 8_78666793 was co-detected for DTS by both BLINK and FarmCPU models. SNP 3_198946071 was co-detected for DTP by the BLINK model and DTT by FarmCPU model. SNP 9_146646966 was co-detected for DTP and DTS by the FarmCPU model. SNP 9_152140631 was detected for all three-flowering time-related traits with two models.

### 3.5. Annotation of Candidate Genes

Referring to the B73RefGen_v4 genome, nine candidate genes associated with DTT, fifteen with DTP, and seven with DTS were identified (Table 4). The function of candidate genes was annotated, and only two genes have unknown functions. Five candidate genes were found to be simultaneously related to multiple traits. The SNP 3_198946071, associated with DTT and DTP, was located within the coding region of Zm00001d043406. Zm00001d047969 and Zm00001d047968 were located in the LD interval of SNP 9_146646966, concurrently affecting DTP and DTS traits. The most crucial SNP 9_152140631, controlling all three-flowering time-related traits, was located in the LD interval of Zm00001d048190 and Zm00001d048191.

### 3.6. Genomic Selection for Flowering Time-Related Traits

The GS prediction accuracies estimated by a 5-fold cross-validation with all the SNPs were 0.47, 0.55, and 0.55 for DTT, DTP, and DTS, respectively.

**Figure 4:**
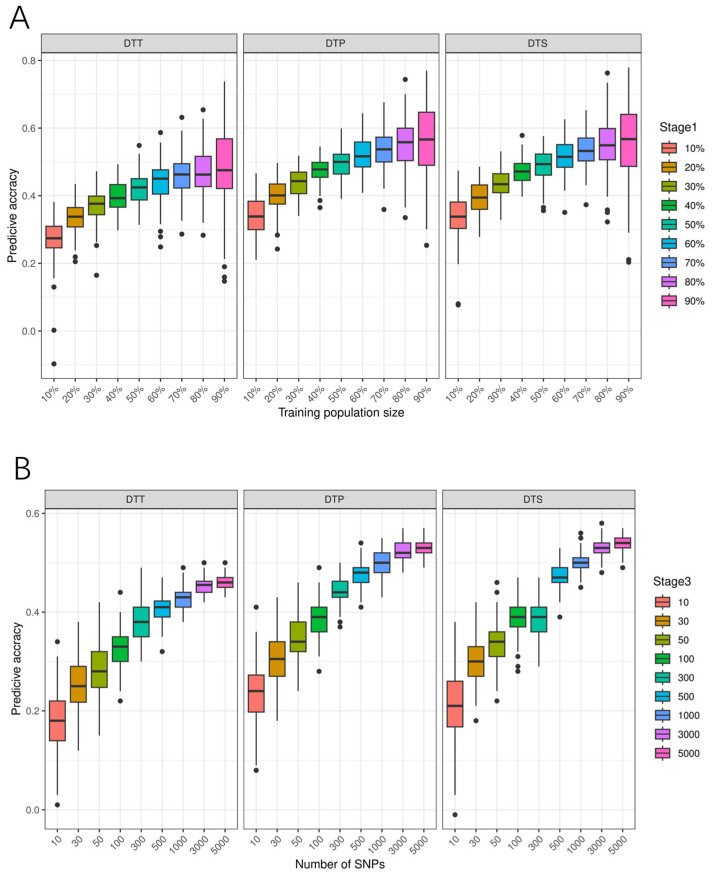

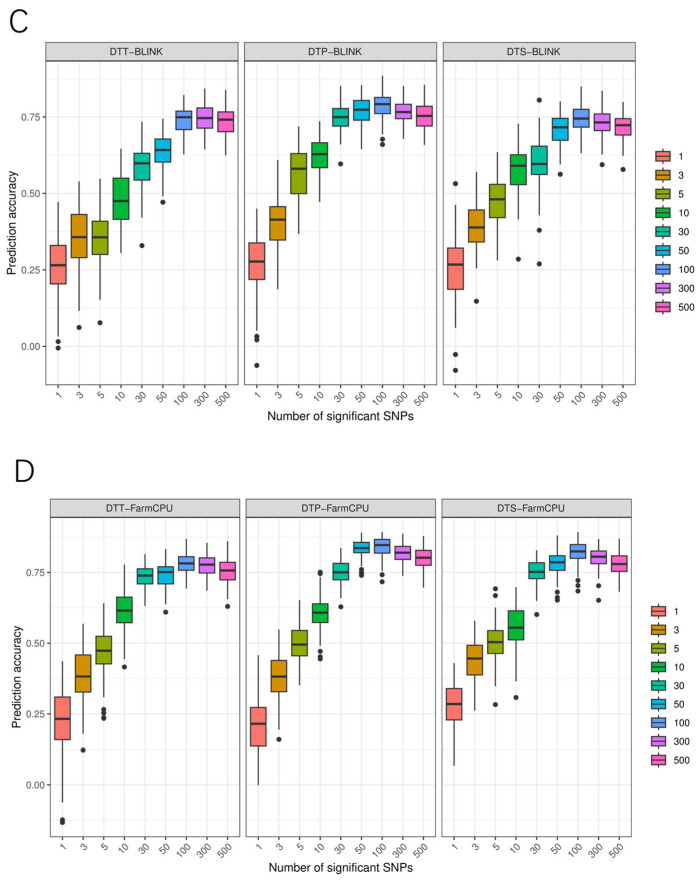
Genomic prediction accuracy of flowering time-related traits. (**A**) the effect of different training population size on prediction accuracy; (**B**) the effect of marker density on prediction accuracy; (**C**) the effect of different number of significant markers on prediction accuracy of GS in BLINK models; (**D**) the effect of different number of significant markers on prediction accuracy of GS in FarmCPU models.

The training population size has a noticeable impact on the prediction accuracy of GS (Figure 4A, Supplementary Table S2). As the training population size increased, the prediction accuracy of GS for flowering time-related traits gradually improved. When the training population size increased to 70%, the prediction accuracy reached a plateau, where the prediction accuracy rose from 0.27 to 0.46 for DTT, from 0.34 to 0.54 for DTP, and from 0.34 to 0.54 for DTS.

The effect of marker density on the prediction accuracy of GS is shown in Figure 4B and Supplementary Table S3. A higher marker density leads to a higher prediction accuracy. When marker density reached 3000, the prediction accuracy of all three traits reached a plateau, 0.46 for DTT, 0.52 for DTP, and 0.53 for DTS.

As the number of SNPs with the lowest *p*-value increased from 1 to 1000, the prediction accuracy first increased and then decreased (Figure 4C,D, Supplementary Table S4). When the number of significant SNPs increased to 100, the highest prediction accuracy was achieved. For the Blink model, the highest prediction accuracies for DTT, DTP, and DTS were 0.73, 0.79, and 0.74, respectively. For the FarmCPU model, the highest prediction accuracies for DTT, DTP, and DTS were 0.78, 0.84, and 0.82, respectively. The FarmCPU model attains higher prediction accuracy than the Blink model. Compared to GS with all the markers, the prediction accuracy using 100 significant markers can be greatly improved.

## 4. Discussion

### 4.1. Genetic Basis of Flowering Time-Related Traits in Maize

Maize has become one of the most widely planted crops in the world [26], mainly because it adapts to different geographical environments via flowering time regulation [27]. The flowering period is a crucial stage in the growth and development of maize. In this study, broad phenotypic variation in DTT, DTP, and DTS was observed in each environment. The heritability of the three-flowering time-related traits was relatively high, indicating that phenotypic selection in the early stage was effective. Two GWAS models were employed to explore the genetic basis of flowering time-related traits in maize. A total of 14 SNPs significantly associated with flowering time-related traits were detected on chromosomes 1, 3, 8, 9, and 10. Three SNPs exhibited a pleiotropic effect. The SNP 9_146646966 was significantly associated with both DTP and DTS. The SNP 3_198946071 was significantly associated with DTT and DTP. One SNP 9_152140631 was identified for all three traits by both models and the PVE ranged from 5.82% to 24.17%. It is inferred that the three-flowering time-related traits might share the same genetic control and possess a similar genetic foundation.

Numerous QTLs associated with maize flowering time-related traits have been localized, and a comprehensive analysis of these results indicates a tendency for flowering QTLs to be distributed on chromosomes 1, 3, 8, 9, and 10 [16,24,27,28,29,30]. Some SNPs detected in the present study were reported by previous studies. The SNP 9_152140631 detected for all three-flowering time-related traits by both models was close to SNP 9_149039896 <num_29 kb (referring to the B73RefGen_v2 genome), which was co-located for DA and DS in a natural association panel (Ames) with 1745 inbred lines from the USDA-ARS NCRPIS [8]. It was in the gene region of Zm00001d048190 and linked to Zm00001d048191. ZM00001D048190 encoded RNase L inhibitor protein-related. Zm00001d048191 encoded a membrane protein. SNP 8_77014638 for DTP and SNP 8_78666739 for DTS were in the same region of *qDPSRS-8-1*, a QTL identified for DTS by single-environment analysis [24]. SNP 8_114509981 for DTP was close to SNP S8_112412901, which was significantly associated with ASI [16]. SNP 10_146767704 and SNP 10_149653921 for DTT, and SNP 10_145158286 for DTP were located at bin 10.07 and in the same region of *qDPSFS-10-1* and *qDPSFJ-10-1* for DPS and *qDTSFJ-10-1* for DTS reported by Liu et al. [24]. The SNP 9_146646966 co-located for DTP and DTS was close to SNP S9_144119131, which was identified for DTS in a panel of 258 tropical maize inbred lines [16]. SNP 1_190275500 detected at bin 1.06 for DTT was in the same interval of *qdsilk13* and *qdsilk45* mapped for DTS [27,28]. SNP 9_146646966 significantly associated with DTP and DPS was mapped in the same region of *qdsilk16* and *qdsilk43* [27,29]. SNP 3_198946071 for both DTT and DTP located at bin3.07 is consistent with the observations of Wang et al. [30]. The SNPs detected by different studies were stable loci controlling flowering time-related traits and some exhibited pleiotropic effects. Developing functional markers for marker-associated selection would effectively improve the breeding efficiency of flowering time-related traits.

### 4.2. Candidate Gene Analysis

Based on the B73 RefGen_v4 genome, candidate genes were screened within 10 Kb upstream and downstream of the 14 significantly associated SNPs for flowering-related traits. A total of 24 candidate genes were identified, of which 22 have functional annotations. Several candidate genes are known to be associated with flowering time. Zm00001d045323 encodes a double B-box zinc finger protein 11. B-box (BBX) proteins play a crucial role in plant growth regulation and development, including photomorphogenesis, photoperiodic regulation of flowering, and responses to biotic and abiotic stresses [31]. The transcription factor BBX is well-known for regulating flowering, photoperiod sensitivity, photomorphogenesis, and stress tolerance [32]. Studies have shown that AtBBX1, the first identified and characterized protein in the BBX family, plays a vital role in regulating flowering time and flower development [33]. In chrysanthemums, CmBBX24 has a dual function, delaying flowering and increasing cold or drought resistance [34]. Zm00001d009708 encodes a calcium-dependent protein kinase (CPK) family protein. A previous study has indicated that the CPK32 gene can control the growth of pollen tubes in tobacco and maize [35]. Plant growth and differentiation depend on the continuous functionality of meristematic tissues. The gene Zm00001d031444 encodes an IRK-interacting protein. It has been reported that the IRK-interacting protein is involved in maintaining and differentiating inflorescence meristematic tissues [36]. The IRK gene is expressed in proliferating and expanding tissues, such as stem meristematic tissues, floral buds, and root meristematic tissues [37]. In flowering plants, the formation of organs, like leaves and flowers, depends on the apical meristematic tissues of shoots [38].

Numerous studies have shown that various plant hormones are involved in regulating floral transition [39,40,41,42]. *ETHYLENE*(*ETH*) *INSENSITIVE3/EIN3-LIKE1* modulates *FLOWERING LOCUS C* expression via histone demethylase interaction [43]. Zm00001d031445 encodes an *ETHYLENE INSENSITIVE3-LIKE* transcription factors that activate the ETH signaling pathway [44]. Zm00001d026397 encodes a protein with an RNA-binding domain, and its homolog in Arabidopsis thaliana (AT2G41060) is functionally annotated to be associated with the ethylene (ETH) biosynthesis processes [45,46]. Zm00001d039650, encoding *Cytochrome P450 734A1*, participates in brassinosteroid (BR) biosynthesis, influencing the flowering of Luculia gratissima and Mikania micrantha [47,48]. It is speculated that these genes affect the expression of flowering time-related traits by participating in hormone metabolism pathways.

According to the gene expression data of 23 tissues spanning vegetative and reproductive stages of maize [49], ZM00001d048190, ZM00001d043406, and ZM00001d047969 have the highest expression in female spikelet collected during the day compared with other samples of B73. These results indicate that ZM00001d048190, ZM00001d043406, and ZM00001d047969 are highly likely to be involved in regulating maize flowering time. These potential candidate genes provide a better understanding of the genetic basis of flowering time-related traits.

### 4.3. Factors Affecting Genomic Selection Prediction Accuracy

Genomic selection can improve breeding efficiency by capturing both minor and major QTLs of the target trait. The GS of flowering time-related traits has been reported in several studies [10,17,43]. The prediction accuracy was 0.64 for the anthesis date and 0.40 for ASI in 300 tropical and subtropical maize inbred lines [10]. These findings were consistent with our study. The prediction accuracy was 0.47 for DTT, 0.55 for DTP, and 0.55 for DTS using the 5-cross validation method.

Previous studies have shown that as the training population size increased, the prediction accuracy of GS rapidly improved and gradually stabilized [50,51]. Liu et al. (2023) showed that when the training population size increased from 60% to 90%, the increase in prediction accuracy of husk tightness could be negligible [52]. Our results showed a similar trend, where relatively high prediction accuracy was achieved with a training population size of 70%.

One of the factors limiting the application of genomic selection is the cost of genotyping. Our study showed that a good prediction accuracy of all three traits was obtained with 3000 SNPs, 0.46 for DTT, 0.52 for DTP, and 0.53 for DTS. Similar results were reported by previous studies. When the number of markers reached 5000, the prediction accuracy of common rust resistance reached a plateau in a GWAS panel [53]. These results can significantly reduce the cost of genotyping and promote the application of GS in breeding programs.

In 2014, Bernardo proposed a new approach for GS that integrates known marker-trait associations in prediction models to improve prediction accuracy [54]. In 2016, Spindel et al. improved the prediction accuracy by incorporating significantly associated loci detected by GWAS into the GS model [55]. The experimental results of Yang et al. in 2017 also demonstrated that incorporating markers with evolutionary information into GS models helps unravel the genetic basis of traits [56]. Liu et al. conducted GWAS and GS of *Fusarium* ear rot resistance in maize using CIMMYT germplasm. A relatively high prediction accuracy was obtained when integrating significantly associated SNPs detected from GWAS in GS [57]. In the DTMA panel, Yuan et al. [10] reported that the prediction accuracies evaluated by significant SNPs identified from GWAS were higher than those evaluated by all the SNPs for flowering time-related traits. In the present study, the prediction accuracy using 100 SNPs with the lowest *p*-value can be greatly improved compared to GS with all the markers. It is not that the higher the number of markers, the better the prediction effect [58]. Too many markers will not only bring great computational pressure but also reduce computational efficiency. Our study provides a theoretical basis for the application of GS in maize breeding.

## 5. Conclusions

In this study, the flowering time-related traits of the association population in maize exhibit rich phenotypic variation, with a relatively high genetic heritability, and strong positive correlations among these traits.

Through a dual-model whole-genome association analysis of maize flowering time-related traits, 14 significant SNP loci associated with the flowering period and 24 potential candidate genes closely related to flowering were detected. These loci play a crucial role in the genetic expression of flowering time-related traits.

Through genomic selection, it was found that the optimal training population size for three-flowering time-related traits is 70%, with a marker density of 3000 SNPs, resulting in high prediction accuracy for all three-flowering traits. When significant associated loci identified by two GWAS methods were incorporated into GS prediction, it significantly improved the accuracy of the prediction models. The significant loci identified by the FarmCPU model are more suitable for the genomic selection of flowering traits.

## Figures and Tables

**Table 1:** Statistical analysis of flowering time-related traits in multiple environments.

Trait ^1^	Environment ^2^	Range	Mean ± SD	SKewness	Kurtosis	CV (%) ^3^
DTT	SG	50.0~72.0	59.94 ± 4.22	0.23	−0.68	7.04
	DF	50.0~62.0	54.63 ± 2.47	0.44	−0.82	4.53
	LD	50.0~68.0	56.41 ± 3.79	0.62	−0.48	6.71
	QT	63.0~83.0	71.06 ± 3.48	0.00	0.29	4.90
	Combined	55.1~67.0	60.55 ± 2.27	0.36	−0.42	3.75
DTP	SG	54.0~77.0	64.30 ± 4.20	0.33	−0.35	6.53
	DF	53.0~75.0	62.80 ± 4.67	0.29	−0.77	7.43
	LD	52.0~72.0	59.80 ± 3.71	0.44	−0.31	6.21
	QT	63.0~86.0	72.90 ± 3.41	0.16	0.70	4.39
	Combined	58.2~73.0	65.00 ± 2.85	0.36	−0.46	4.67
DTS	SG	54.0~78.0	65.87 ± 4.51	0.27	−0.44	6.85
	DF	54.0~78.0	63.73 ± 5.45	0.42	−0.88	8.55
	LD	52.0~74.0	61.29 ± 3.91	0.31	−0.08	6.39
	QT	63.0~87.0	73.78 ± 3.78	0.78	0.80	5.13
	Combined	58.8~75.0	66.30 ± 3.16	0.39	−0.47	4.76

^1^ Trait: DTT: days to tasseling; DTP: days to pollen-shedding; DTS: days to silking. ^2^ SG: Sangong Town experimental station, Changji City, Xinjiang, China; DF: Dafeng Town experimental station, Hutubi County, Xinjiang, China; LD: Ledong experimental station, Hainan Province, China; QT: Qitai County experimental station, Xinjiang, China; Combined: A combination of four environments. ^3^ CV: coefficient of variation.

**Table 2:** Variance analysis of flowering time-related traits.

Trait ^1^	Variance Components ^2^			*H*^2^ (%) ^3^
	${\hat{\sigma }}_{g}^{2}$	${\hat{\sigma }}_{ge}^{2}$	${\hat{\sigma }}_{e}^{2}$	
DTT	13.61 ***	9.22 ***	4.26 ***	82.75
DTP	19.84 ***	10.68 ***	4.29 ***	86.09
DTS	23.98 ***	14.12 ***	4.92 ***	85.26

^1^ DTT: days to tasseling; DTP: days to pollen-shedding; DTS: days to silking. ^2^ ${\hat{\sigma }}_{g}^{2}$: genotype variance; ${\hat{\sigma }}_{ge}^{2}$: genotype × environment interaction variance; ${\hat{\sigma }}_{e}^{2}$: error variance; ***: significant at the 0.001 level. ^3^ *H*^2^: broad-sense heritability.

**Table 3:** Significant SNPs for flowering time-related traits.

Model	Trait ^1^	SNP ^2^	Chromosome	Position	Bin	*p*-Value	R^2^ (%) ^3^
BLINK	DTT	1_190275500	1	190275500	1.06	5.52 × 10^−11^	1.41
		9_152140631	9	152140631	9.07	2.48 × 10^−9^	16.92
		10_146767704	10	146767704	10.07	2.34 × 10^−8^	1.97
	DTP	3_198946071	3	198946071	3.07	3.23 × 10^−8^	2.64
		3_229665314	3	229665314	3.09	2.68 × 10^−7^	3.90
		8_114509981	8	114509981	8.04	2.26 × 10^−7^	3.70
		9_152140631	9	152140631	9.07	3.98 × 10^−11^	12.68
	DTS	8_78666793	8	78666793	8.03	2.42 × 10^−12^	3.39
		9_152140631	9	152140631	9.07	3.52 × 10^−7^	24.17
FarmCPU	DTT	3_198946071	3	198946071	3.07	2.52 × 10^−8^	4.34
		9_152140631	9	152140631	9.07	7.35 × 10^−15^	15.53
		10_149653921	10	149653921	10.07	1.27 × 10^−7^	3.55
	DTS	3_10537234	3	10537234	3.03	2.67 × 10^−8^	1.02
		8_77014638	8	77014638	8.03	2.05 × 10^−7^	0.50
		9_18946215	9	18946215	9.02	1.42 × 10^−7^	3.74
		9_146646966	9	146646966	9.06	3.90 × 10^−9^	1.18
		9_152140631	9	152140631	9.07	8.05 × 10^−13^	5.82
		10_145158286	10	145158286	10.07	7.89 × 10^−14^	3.73
	DTS	3_199485386	3	199485386	3.07	1.35 × 10^−7^	7.16
		8_78666793	8	78666793	8.03	8.63 × 10^−9^	2.30
		9_146646966	9	146646966	9.06	3.68 × 10^−7^	3.30
		9_152140631	9	152140631	9.07	3.40 × 10^−9^	8.53

^1^ DTT: days to tasseling; DTP: days to pollen-shedding; DTS: days to silking. ^2^ SNP name, chromosome position. ^3^ R^2^: the phenotypic variance explained by each SNP.

**Table 4:** Putative candidate gene of flowering time-related traits.

SNP	Trait	Gene ID	Annotation
1_190275500	DTT	Zm00001d031445	Ethylene-insensitive3-like protein
		Zm00001d031444	IRK-interacting protein
		Zm00001d031443	DUF2361 family protein
		Zm00001d031447	ABC transporter C family member 10
3_10537234	DTP	Zm00001d039650	Cytochrome P450 734A1
3_198946071	DTT, DTP	Zm00001d043406	Interactor of constitutive active ROPs 1
3_199485386	DTS	Zm00001d043427	Uncharacterized LOC109945323
		Zm00001d043426	Pentatricopeptide repeat-containing protein At3g58590
3_229665314	DTP	Zm00001d044495	Vacuolar-processing enzyme
		Zm00001d044496	Pentatricopeptide repeat-containing protein chloroplastic
8_77014638	DTP	Zm00001d009708	Calcium-dependent protein kinase 1
		Zm00001d009707	Protein ABIL1
8_78666793	DTS	Zm00001d009738	Tubulin γ-2 chain
8_114509981	DTP	Zm00001d010425	Coiled-coil domain-containing protein 25
9_146646966	DTP, DTS	Zm00001d047969	CONSTANS interacting protein 6
		Zm00001d047968	Zinc finger protein 7
9_152140631	DTT, DTP, DTS	Zm00001d048190	RNase L inhibitor protein-related
		Zm00001d048191	Membrane protein
9_18946215	DTP	Zm00001d045323	Double B-box zinc finger protein 11
		Zm00001d045324	Dual specificity protein phosphatase DSP8
10_145158286	DTP	Zm00001d026397	RNA-binding protein AKIP1
		Zm00001d026396	Formation of crista junctions protein 1
10_146767704	DTT	Zm00001d026482	Uncharacterized LOC111590308
10_149653921	DTT	Zm00001d026668	Receptor-like kinase TMK2

## Data Availability

The data presented in this study are available in this article and the Supplementary Materials.

## Supplementary Materials

The following supporting information can be downloaded at: https://www.mdpi.com/article/10.3390/genes15060740/s1, Table S1: The 21 maize hybrids used for DH population construction. Table S2: Genomic selection prediction accuracy estimated by different training population sizes. Table S3: Genomic selection prediction accuracy estimated by different marker densities. Table S4: Genomic selection prediction accuracy estimated by all the markers and significant SNPs.
