# Supplementary material for: Dual-Model GWAS Analysis and Genomic Selection of Maize Flowering Time-Related Traits
Source: Genes (Basel). 2024 Jun 4;15(6):740. doi: 10.3390/genes15060740 (PMC11203321; doi:10.3390/genes15060740)
Supplement: Supplementary file 1 [file genes-15-00740-s001.zip › genes-3001697-supplementary.pdf]

Supplementary Table 1 The 21 maize hybrids used for DH population construction.

No.	Name
1	XY1466
2	XY1366
3	XY1266
4	XY1148
5	XY1140
6	XY047
7	XY1225
8	XY335
9	XY1224
10	J1652
11	DK653
12	C6361
13	Lidan771
14	Lidan638
15	Lidan618
16	DK516
17	Lihe869
18	DK159
19	ZD958
20	Lidan295
21	JK968

Supplementary Table 2 Genomic selection prediction accuracy estimated by different training population sizes.

Trait	Training population size	Prediction accuracy
DTT	90%	0.48a
	80%	0.47ab
	70%	0.46ab
	60%	0.44bc
	50%	0.42cd
	40%	0.40de
	30%	0.37e
	20%	0.33f
	10%	0.27g
DTP	90%	0.56a
	80%	0.55a
	70%	0.54ab
	60%	0.52bc
	50%	0.50cd
	40%	0.47d
	30%	0.44e
	20%	0.40f
	10%	0.34g
DTS	90%	0.56a
	80%	0.55a
	70%	0.54ab
	60%	0.51bc
	50%	0.49cd
	40%	0.47d
	30%	0.44e
	20%	0.40f
	10%	0.34g

Note: Different letters mean significant differences ($P < 0.05$). The same letters mean no significant differences ($P \geq 0.05$).

Supplementary Table 3 Genomic selection prediction accuracy estimated by different marker density.

Trait	Number of SNPs	Prediction accuracy
DTT	5000	0.46a
	3000	0.46a
	1000	0.43b
	500	0.41c
	300	0.38d
	100	0.39e
	50	0.37f
	30	0.36g
	10	0.32h
DTP	5000	0.53a
	3000	0.52a
	1000	0.50b
	500	0.47c
	100	0.46d
	300	0.44e
	50	0.44f
	30	0.42g
	10	0.38h
DTS	5000	0.53a
	3000	0.53a
	1000	0.50b
	500	0.47c
	300	0.38d
	100	0.46d
	50	0.44e
	30	0.42f
	10	0.37g

Note: Different letters mean significant differences($P < 0.05$). The same letters mean no significant differences ($P \geq 0.05$).

Supplementary Table 4 Genomic selection prediction accuracy estimated by all the markers and significant SNPs.

Trait	Group	Prediction accuracy
DTT	100 significant SNPs of FarmCPU	0.78a
	100 significant SNPs of Blink	0.74b
	all markers	0.47c
DTP	100 significant SNPs of FarmCPU	0.83a
	100 significant SNPs of Blink	0.79b
	all markers	0.55c
DTS	100 significant SNPs of FarmCPU	0.82a
	100 significant SNPs of Blink	0.74b
	all markers	0.55c

Note: Different letters mean significant differences($P < 0.05$). The same letters mean no significant differences ($P \geq 0.05$).
